# Case Report: Cystic fibrosis transmembrane conductance regulator gene heterozygous variation presenting with abdominal pain and hepatopancreatic lesions in a child

**DOI:** 10.3389/fped.2025.1707993

**Published:** 2026-02-05

**Authors:** Xiu Lu, Lidong Ning, Hong Zhen, Ming Liang, Yulan Han, Hongyan Wei, Lingdong Zeng, Lihong Wei, Liqin Tan

**Affiliations:** 1 Department of Pediatrics, The Second Affiliated Hospital of Guangxi Medical University, Nanning, Guangxi, China; 2 Department of Neurosurgery, National Hospital of Guangxi Zhuang Autonomous Region, Nanning, Guangxi, China; 3 Emergency Department, The Second Affiliated Hospital of Guangxi Medical University, Nanning, Guangxi, China

**Keywords:** child, cystic fibrosis, cystic fibrosis transmembrane conductance regulator, hepatopancreatic ampulla, pancreatitis

## Abstract

**Background:**

Cystic fibrosis (CF) is an autosomal recessive disorder caused by mutations in the cystic fibrosis transmembrane conductance regulator (CFTR) gene, leading to multi-system involvement.

**Case report:**

A 4-year-old girl presented with a 2-day history of abdominal pain. Clinical manifestations included recurrent epigastric pain and vomiting. Physical examination revealed abdominal distension, mild periumbilical tenderness, and hepatomegaly (liver palpable 4 cm below the right costal margin). Laboratory tests showed elevated pancreatic enzymes: serum amylase 607 U/L, lipase 634 U/L, and pancreatic amylase 252 U/L (all >3 times the upper limit of normal). Abdominal ultrasound demonstrated diffuse hepatic lesions and uneven echogenicity in pancreatic parenchyma. Computed tomography revealed chronic liver disease changes, possible cirrhosis and a slightly enlarged spleen. Hepatobiliary histopathological biopsy indicated biliary obstruction. Whole-exome sequencing identified CFTR allele variants c.3139G > T (paternal source) and c.1409T > A (maternal source). Comparative analysis with the existing literature verified that the G > T mutation at chromosome 7 (chr7):117250723 was previously unreported. Treatment with octreotide, omeprazole, and pancreatic enzyme replacement therapy led to symptom resolution. At follow-up, her condition remained stable: height=109 cm (10th to 25th percentile), weight=18.8 kg (25th to 50th percentile) and stable condition.

**Conclusion:**

The clinical manifestations of CF are diverse, and digestive tract symptoms are common; therefore, early identification and diagnosis are required. As chr7: 117250723 G > T may be a pathogenic gene, long-term follow-up is needed.

## 1 Background

Cystic fibrosis (CF) is an autosomal recessive genetic disorder affecting children's health. The disease mechanism stems from mutations in the CF transmembrane conductance regulator (*CFTR*) gene, which disrupts the function of a channel protein. Located at 7q31.2 on human chromosome 7 (chr7), the *CFTR* gene encodes a 1,480-amino acid cross-membrane chloride ion channel protein. This protein is widely distributed on the surfaces of epithelial cells in the lungs, pancreas, liver, intestines and other organs. Its primary role is to regulate the transmembrane transport of chloride ions and water, maintaining stable mucus viscosity. Mutated channel proteins exhibit structural or functional abnormalities that impair ion–water transport, increase mucus production and cause airway blockage. These pathogenic changes affect respiratory, digestive and reproductive systems, clinically manifesting as recurrent respiratory infections and pancreatic insufficiency (1).

CF incidence is higher in Caucasians (1:25,000–1:1,800) (2). Diagnosis in China remains challenging due to limited availability of sweat chloride testing and high-cost genetic sequencing, resulting in underdiagnosis and a lack of population-based incidence data despite its inclusion in the national rare disease catalog (3, 4).

Clinical and genotypic profiles differ between populations. In Caucasians, the F508del mutation predominates (∼70%), with progressive lung disease as the hallmark. Chinese patients exhibit diverse genotypes and more frequent gastrointestinal involvement (4).

## 2 Case data

### 2.1 Admission Status

A 4-year-old female patient was admitted in May 2023 with abdominal pain that had lasted for 2 days. Two days prior, she had developed persistent dull pain around the navel without apparent cause, accompanied by vomiting of gastric contents twice (non-projectile, non-haemorrhagic and coffee-ground-like). No fever, convulsions, abdominal distension or diarrhoea were present. Laboratory tests at other hospitals revealed normal routine bloods, liver function and hepatitis B serology panel. Abdominal ultrasound revealed diffuse liver lesions. The child had no notable medical history, medication allergies, surgical trauma or family history of similar conditions, and a detailed medical history revealed that the child was previously healthy without any history of recurrent respiratory infections or chronic abdominal pain. Dietary history was unremarkable, with no known food allergies or specific dietary restrictions. Computed tomography (CT) suggested potential liver abnormalities. She was transferred to our hospital for further evaluation.

### 2.2 Admission for medical examination

Vital signs were stable: temperature 36.8 °C, heart rate 108 bpm, respiratory rate 22/min, blood pressure 96/62 mmHg. Her height was 107 cm [25th to 50th percentile (P25–50)], and her weight was 17.9 kg (P50). She was conscious and alert and had normal facial features. No jaundice, rash, or lymphadenopathy was noted. Cardiopulmonary examination was normal. The abdomen was distended. The patient experienced mild tenderness around the umbilicus, but the lower abdomen was otherwise non-tender and non-remitting. The liver was palpable 4 cm below the right costal margin (moderate consistency with blunt margins), but the spleen was not detected. Bowel sounds were normal (4/min). The extremities were warm with a 2-second capillary refill time. Neurological examination showed no abnormalities.

### 2.3 Accessory examination

Blood tests on admission showed elevated C-reactive protein (12.21 mg/L; normal <10 mg/L) but normal white cell count, hemoglobin, and platelets (Table 1). Pancreatic enzymes were markedly elevated: serum amylase 250 U/L (normal 28-100), lipase 230 U/L (normal 13-60), pancreatic amylase 228 U/L (normal 13-53) (Table 2). The peak levels of serum amylase (607 U/L), lipase (634 U/L), and pancreatic amylase (252 U/L) observed during hospitalization are summarized in the Abstract and detailed in Table 2. Liver function tests on admission were notable for elevated total bile acids (19.7 μmol/L; normal 0–10) (Table 3).

**Table 1:** Dynamic changes in hematological parameters and hypersensitive C-reactive protein during hospitalization and follow-up.

Parameter	5–13	5–15	5–25	6–8	8–7	Reference ranges
White blood cell count (WBC, × 10⁹/L)	9.09	4.33	9.07	6.29	9.23	4–10 × 10⁹/L
Hemoglobin (Hb, g/L)	127.00	115.00	135.00	118.00	120.00	110–150 g/L
Platelet count (PLT, × 10⁹/L)	262.00	202.00	304.00	217.00	238.00	100–300 × 10⁹/L
Neutrophils (Neut, %)	0.701	0.439	0.651	0.553	0.513	50%–70%
Lymphocytes (Lym, %)	0.196	0.381	0.260	0.313	0.358	20%–40%
Monocytes (Mono, %)	0.100	0.127	0.039	0.091	0.051	3%–8%
Lymphocyte count (Lym abs, × 10⁹/L)	1.78	1.65	2.36	1.97	3.30	0.8–4 × 10⁹/L
C-reactive protein (hs-CRP, mg/L)	12.21	22.29	0.70	3.72	1.45	0–10 mg/L

Dates in the table represent time points during hospitalization and follow-up (month-day). All parameters were determined by standard automated hematology analyzers. hs-CRP was measured by immunoturbidimetry. Reference ranges are based on the standards of our hospital's pediatric laboratory.

WBC, white blood cell; Hb, hemoglobin; PLT, platelet; Neut, neutrophils; Lym, lymphocytes; Mono, monocytes; abs, absolute; hs-CRP, high-sensitivity C-reactive protein.

**Table 2:** Serial monitoring of Serum pancreatic enzymes during hospitalization (unit: U/L).

Parameter	5–13	5–15	5–17	5–19	5–21	5–23	5–25	5–28	6–5	7–23	8–7	Reference ranges
Serum lipase	230	190	119	233	310	528	596	634	–	–	200	13–60 U/L
Serum amylase	250	185	51	63	144	259	267	240	607	166	137	28–100 U/L
Pancreatic amylase	228	170	43	54	131	249	252	224	–	–	118	13–53 U/L

Dates in the table represent time points during hospitalization and follow-up (month-day). All serum enzymatic assays (lipase, amylase, pancreatic amylase) were measured by enzymatic spectrophotometric methods. Reference ranges are based on pediatric standards provided by the reagent manufacturer. The symbol “-” indicates that the test was not performed at that specific time point.

**Table 3:** Comparison of liver function profiles at admission and after treatment during follow-up.

Liver function parameter	2023–5-13	2023-8-7	Reference ranges
Total bilirubin (TBIL, μmol/L)	14.6	3.1	0–17.0 μmol/L
Direct bilirubin (DBIL, μmol/L)	6.5	1.9	0–8.0 μmol/L
Gamma-glutamyl transferase (GGT, U/L)	49	79	10–60 U/L
Total bile acids (TBA, μmol/L)	3.3	19.7	0–10 μmol/L
Aspartate aminotransferase (AST, U/L)	31	51	15–40 U/L
Alanine aminotransferase (ALT, U/L)	23	45	9–50 U/L

Samples were collected at initial presentation (2023-5-13) and during a key follow-up visit after primary treatment (2023-8-7). All liver function parameters were assayed using standard biochemical analyzers. Reference ranges are based on the standards of our hospital's pediatric laboratory.

TBIL, total bilirubin; DBIL, direct bilirubin; GGT, gamma-glutamyl transferase; TBA, total bile acids; AST, aspartate aminotransferase; ALT, alanine aminotransferase.

**Figure 1:**
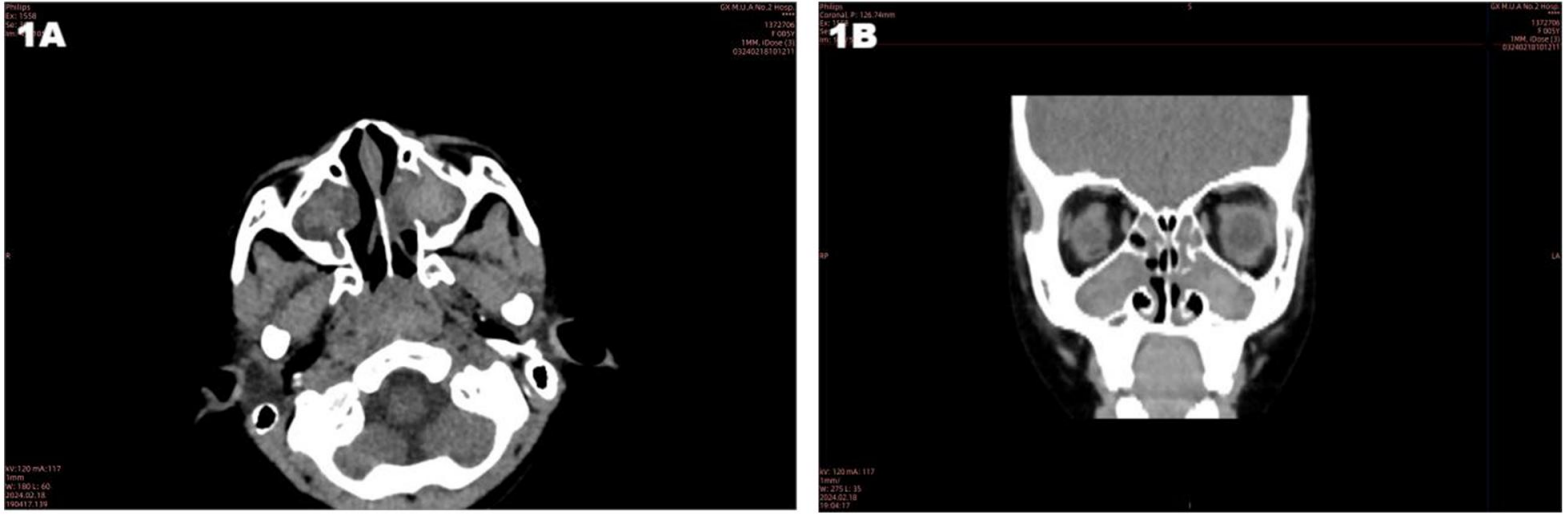
CT of nasal sinuses: sinusitis. **(A)** Coronal view of the paranasal sinuses. The image shows obvious thickening of the mucous membrane in the maxillary sinuses and ethmoid sinuse, with partial narrowing of the sinus ostia-meatal complex. **(B)** Axial view of the paranasal sinuses. It further confirms the presence of sinusitis: the mucous membrane of the frontal sinuses and sphenoid sinuses is slightly thickened, and there is no obvious effusion in the sinus cavity or destruction of the sinus bony structure.

**Figure 2:**
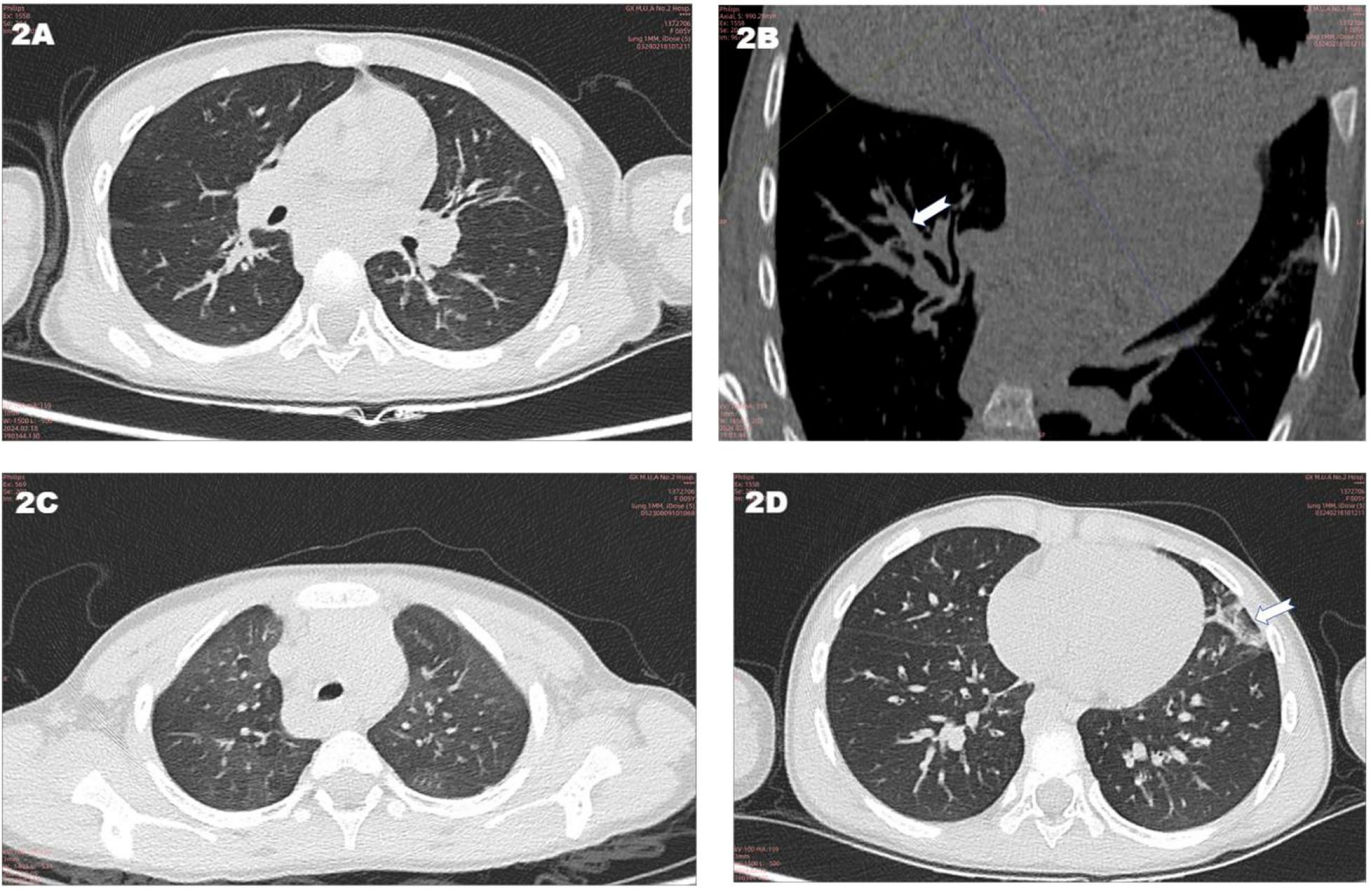
Chest computed tomography (CT) findings: **A** axial CT image of the chest (lung window) shows a well-defined nodular lesion in the left hilum, with no obvious bronchial obstruction, **B** coronal reconstructed CT image (mediastinal window) reveals focal stenosis and irregularity of the right middle lobe bronchus (arrow), suggesting bronchial involvement, **C** axial CT image of the upper chest (lung window) demonstrates multiple small nodular opacities bilaterally, predominantly in the peripheral lung zones, **D** axial CT image of the lower chest (lung window) shows patchy ground-glass opacities and small nodules in the left lower lung, with a focal area of consolidation (arrow).

**Figure 3:**
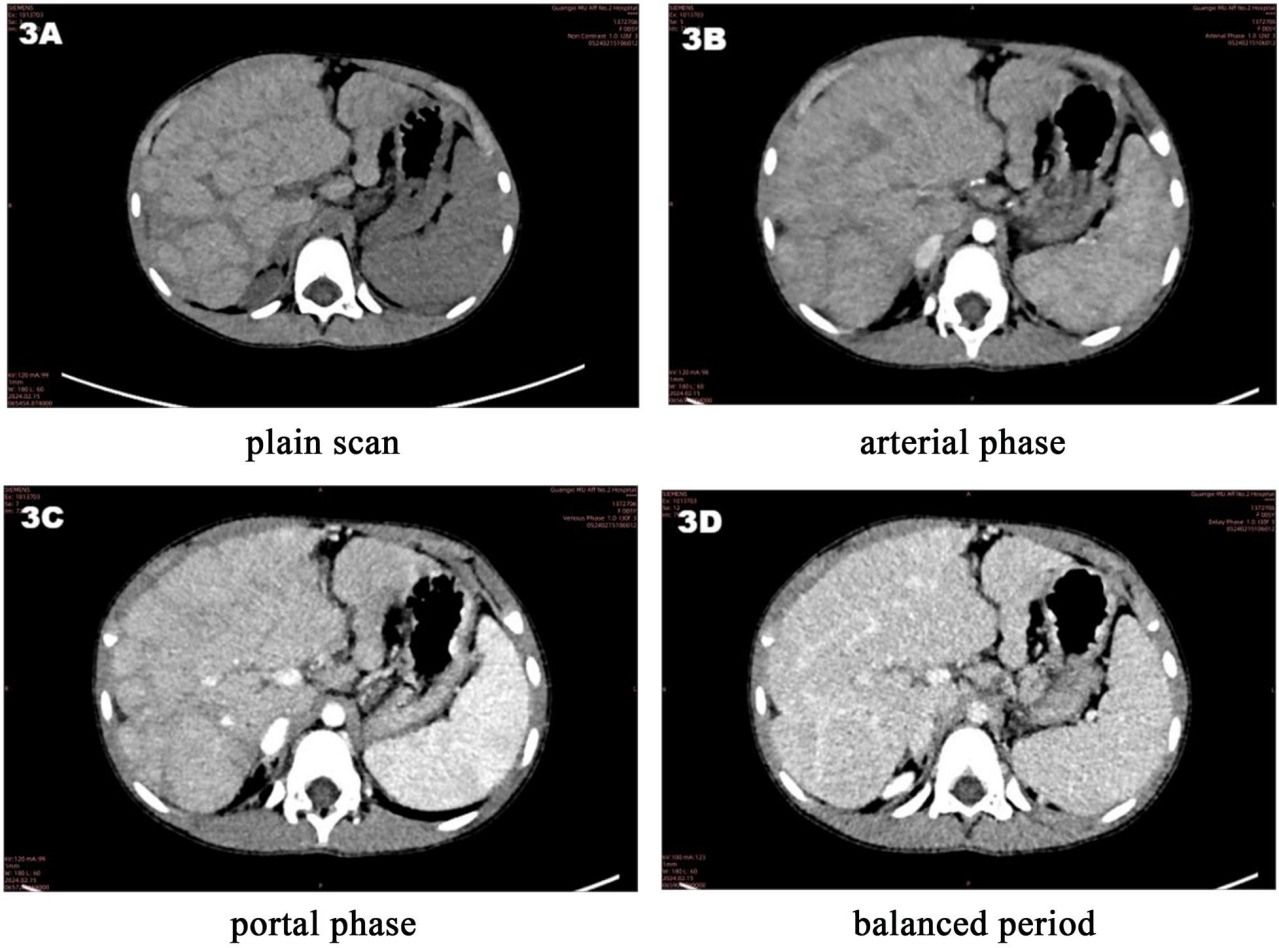
Full abdominal CT plain scan + enhancement: cirrhosis, splenomegaly. **(A)** Plain scan of the abdomen. The image shows irregular contour of the liver, reduced volume of the right hepatic lobe, and increased density of the liver parenchyma (marked by white arrows), which are typical imaging signs of early cirrhosis. **(B)** Arterial phase of abdominal enhanced CT. It shows uneven enhancement of the liver parenchyma. **(C)** Portal phase of abdominal enhanced CT. The portal vein and its branches (e.g., left and right portal veins) are clearly visualized, with no obvious stenosis or thrombosis. **(D)** Balanced period of abdominal enhanced CT. The liver parenchyma shows relatively uniform enhancement (compared with the arterial phase), and the boundary between the normal and fibrotic areas becomes clearer.

Imaging findings: Sinus CT showed sinusitis (Figure 1). Chest CT revealed bronchial wall thickening, a mucus plug in the right lower lobe, mosaic attenuation, and left upper lobe atelectasis (Figure 2). Abdominal CT demonstrated diffuse hepatic lesions, heterogeneous pancreatic texture, and splenomegaly (Figure 3). Notably, liver elastography was not performed in this case. The primary reasons were that abdominal ultrasound and CT had already clearly indicated diffuse hepatic lesions and fibrotic tendencies, and the liver biopsy had provided definitive pathological evidence of fibrosis (see pathological results of liver biopsy), which is considered a reference standard for evaluating liver fibrosis. Consequently, the additional diagnostic value of liver elastography was deemed limited in the clinical decision-making at that time.

**Figure 4:**
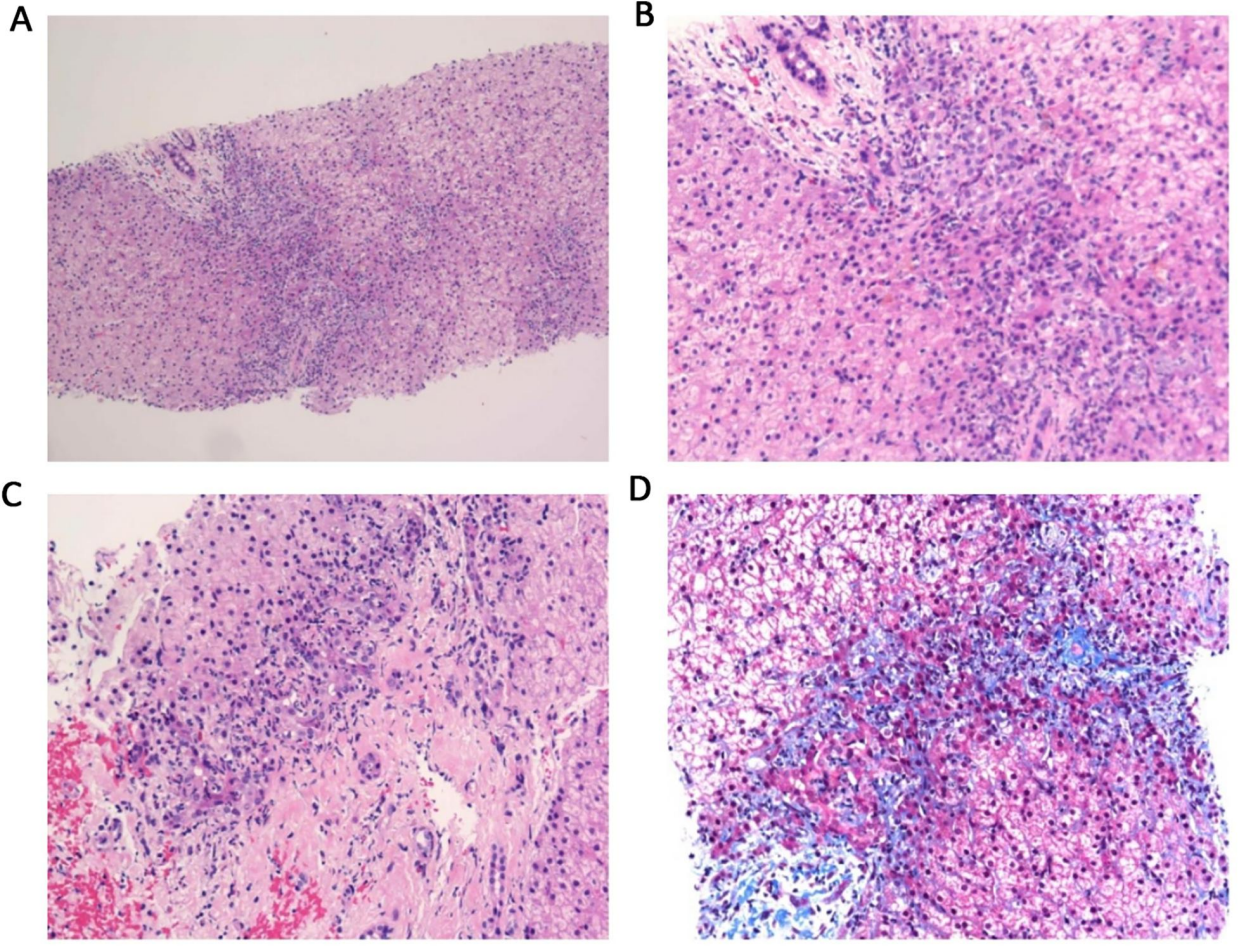
Pathological findings. **(A)** Low-power view (hematoxylin and eosin, H&E staining, × 100) shows diffuse infiltration of inflammatory cells and structural disruption of lung tissue. **B**: High-power view (H&E staining, × 200) reveals a dense infiltrate of histiocytes, lymphocytes, and plasma cells, with no obvious malignant cells. **C**: High-power view (H&E staining, × 200) demonstrates focal hemorrhage (red areas) and mixed inflammatory cell infiltration. **D**: Special staining (Masson trichrome, × 200) shows extensive fibrosis (blue areas) interspersed with inflammatory cells, indicating chronic inflammatory changes with fibrotic progression.

Pathological examination: Haematoxylin and eosin and Masson staining showed moderate-to-severe oedema, mild punctate necrosis and fibrous tissue hyperplasia as well as lymphocyte, neutrophil and eosinophil infiltration in the hepatocytes (Figure 4).

Due to limitations in our hospital's testing capabilities, referral for faecal elastase-1 testing to objectively assess pancreatic exocrine function was recommended but was declined by the parents for personal reasons. Given the biochemical and imaging evidence of pancreatic involvement and the clinical suspicion of malabsorption, pancreatic enzyme replacement therapy was initiated during hospitalization for both acute management and anticipated long-term pancreatic exocrine support.

### 2.4 Genetic sequencing

The study employed whole-exome sequencing technology through the following protocol: peripheral genomic DNA was extracted from both the affected child and her parents, followed by exome capture using the Agilent SureSelect Human All Exon V6 kit. High-throughput sequencing was performed on the Illumina NovaSeq 6000 platform, with a coverage depth of ≥98%, average sequencing depth ≥100×, target region average depth ≥50× and Q30 quality score of ≥90%. Bioinformatics analysis included sequence alignment (using reference genome hg19), variant detection and annotation to identify potential pathogenic *CFTR* gene variants, which were subsequently validated by Sanger sequencing. The results revealed the CFTR allele variants c.3139G > T (paternal source) and c.1409T > A (maternal source), with the G > T mutation at position chr7:117250723 being the first reported case. The Sanger sequencing results of the *CFTR* gene mutations in the patient and her parents, verifying the parental source of the two heterozygous variants, are shown in Supplementary Figure S1.

### 2.5 Differential diagnosis

The diagnostic process for this case required differentiation from the following main conditions:

Acute pancreatitis: The child presented with abdominal pain and vomiting, with serum levels of amylase, lipase and pancreatic amylase all more than three times the upper limit considered normal, meeting the biochemical diagnostic criteria for acute pancreatitis. However, idiopathic acute pancreatitis alone could not adequately explain the concurrent diffuse hepatic lesions and imaging findings suggestive of chronic liver disease. It is noteworthy that specific types of *CFTR* gene mutations are significantly associated with the risk of pancreatitis. Patients with CF carrying “mild” genotypes that preserve pancreatic function have a relatively high risk of developing pancreatitis precisely because they retain functional pancreatic acinar tissue (1). The compound heterozygous variants carried by this patient likely constitute the genetic basis leading to pancreatic duct obstruction, aberrant zymogen activation and subsequent pancreatitis-like manifestations.

Autoimmune liver disease: The child's liver imaging showed diffuse lesions, and histopathology revealed inflammatory cell infiltration, necessitating the exclusion of diseases such as autoimmune hepatitis. However, subsequent testing for relevant autoimmune antibody panels was negative, and there were no other characteristic features of autoimmune diseases, making this diagnosis less likely.

Genetic metabolic liver disease: Given the patient's age and hepatic involvement, genetic metabolic diseases such as alpha-1-antitrypsin deficiency or Wilson's disease were considered. However, systematic genetic metabolic screening and serum marker tests revealed no abnormal evidence, thus rendering such diseases unlikely.

In summary, although the clinical presentation was consistent with acute pancreatitis, the characteristic involvement of multiple organs (pancreas and liver) strongly suggested an underlying systemic aetiology. Combined with the subsequent genetic sequencing identifying compound heterozygous CFTR allele variants, including the novel c.3139G > T variant, the diagnosis of CF with associated hepatopancreatic pathology was ultimately supported. This diagnostic approach aligns with recent studies emphasising the need for *CFTR* gene screening in children presenting with multi-system involvement.

### 2.6 Diagnostic basis

In accordance with the *Chinese Expert Consensus on Diagnosis and Treatment of Cystic Fibrosis (2023 Edition)*, the diagnosis in this paediatric case is based on the following aspects, integrating clinical presentation, auxiliary examinations and genetic sequencing results: (1) Clinical manifestations: The child presented with recurrent epigastric pain and vomiting as the main digestive symptoms. Physical examination revealed hepatomegaly (4 cm below the right costal margin). (2) Auxiliary examination evidence: Pancreatic function/damage markers: Serum amylase, lipase and pancreatic amylase levels were all more than three times the upper limit considered normal and were accompanied by a significant elevation in urinary amylase, consistent with the biochemical features of pancreatic involvement. Imaging characteristics: Abdominal ultrasound suggested diffuse hepatic lesions and heterogeneous pancreatic parenchymal echogenicity; abdominal CT suggested chronic liver disease changes and a slightly enlarged spleen; chest CT indicated bronchial wall thickening, mucus plugs and other airway abnormalities; sinus CT indicated sinusitis. These multi-organ imaging abnormalities are highly suggestive of CF. Pathological support: Liver biopsy indicated biliary injury, fibrous tissue hyperplasia and inflammatory cell infiltration, aligning with the pathological changes of CF-associated liver disease (CFLD). (3) Genetic evidence: Whole-exome sequencing identified compound heterozygous variants in the *CFTR* gene: c.3139G > T (paternal origin) and c.1409T > A (maternal origin). Among these, c.3139G > T (p.Gly1047Cys) is a novel variant reported for the first time internationally. According to the American College of Medical Genetics and Genomics guidelines, this variant is classified as likely pathogenic. The c.1409T > A variant (p.Val470Glu) is conditionally pathogenic, and the combination of these two variants constitutes the molecular aetiology of the disease.

In summary, this paediatric case demonstrates characteristic multi-organ involvement of the digestive system (liver, pancreas) and respiratory system (lungs, sinuses) and carries compound heterozygous pathogenic variants in the *CFTR* gene, fulfilling the diagnostic criteria for atypical CF.

### 2.7 Treatment and follow-up

After hospital admission, the patient received continuous intravenous administration of octreotide (2 ug/kg.h) for enzyme inhibition along with a 10-mg omeprazole intravenous drip (once a day) to suppress acid production and was instructed to fast and receive fluid replacement therapy. The treatment regimen was supplemented with 180 mg of oral azithromycin (once a day) for infection control and 0.5 g of *Bifidobacterium* orally (three times a day) to regulate gut flora. Upon discharge, considering the underlying CF-related pancreatic exocrine dysfunction, the patient was prescribed pancreatic enzyme enteric-coated capsules (specification: 0.15 g per capsule) for long-term pancreatic enzyme replacement therapy at a dose of one capsule three times a day, administered between meals.

After 3 days of treatment, the child reported resolution of abdominal pain. Physical examination showed decreased periumbilical tenderness, and no vomiting occurred. Diet was gradually advanced from fasting to liquid foods several days after admission, with no recurrence of abdominal pain-related crying or refusal to eat. Blood amylase levels were 250 U/L (exceeding the reference range) on 13 May, decreasing to 51 U/L (within the reference range) on 17 May and stabilising at 137 U/L (reference range: 28–100 U/L) on 7 August. Lipase levels were 230 U/L on 13 May, decreasing to 119 U/L (within the reference range) on 17 May and stabilising at 200 U/L (reference range: 13–60 U/L) on 7 August. Pancreatic amylase levels were 228 U/L (three times the reference range) on 13 May, decreasing to 43 U/L (within the reference range) on 17 May and stabilising at 118 U/L (reference range: 13–53 U/L) on 7 August. The dynamic changes of these four indicators during hospitalisation are shown in Supplementary Figure S2, which clearly reflects the trend in indicator reduction and stabilisation after treatment.

After 6 months of follow-up, the abdominal pain did not recur, and the liver enzymes (aspartate aminotransferase, alanine aminotransferase) and pancreatic enzymes were stable in the normal range. The patient's height was 109 cm at age 5 (P10–25), with a weight of 18.8 kg (P25–50).

## 3 Discussion

The clinical manifestations of CF are highly heterogeneous and correlate with the severity of mutations in the *CFTR* gene. This case illustrates a heterozygous *CFTR*-related phenotype with predominant gastrointestinal involvement. This phenotypic presentation aligns with the identified compound heterozygous CFTR variants c.3139G > T (p.Gly1047Cys) and c.1409T > A (p.Val470Glu). Pancreatitis risk in CF correlates with residual pancreatic function. Patients with milder genotypes (pancreatic sufficiency) have a higher pancreatitis risk precisely because they retain functional acinar tissue (1, 5–17). Our patient's genotype likely confers intermediate function, explaining the significant enzyme elevation and abdominal pain.

The novel c.3139G > T (p.Gly1047Cys) variant likely disrupts the transmembrane domain, impairing chloride channel function (18, 19). The c.1409T > A (p.Val470Glu) variant may further affect ATP binding (19).The hepatobiliary lesions result from defective chloride/bicarbonate secretion, leading to viscous bile, cholestasis, periductal inflammation, and fibrosis (4, 8, 13, 20, 21).

Furthermore, the diffuse hepatic lesions observed in this case can be explained by the pathophysiological process resulting from CFTR protein dysfunction in the biliary system. The functional defect in *CFTR* leads to reduced secretion of chloride and bicarbonate by cholangiocytes, resulting in viscous bile, impaired bile flow and cholestasis (8). The inspissated bile predisposes to the formation of microscopic plugs, causing partial biliary obstruction, which subsequently triggers periductal inflammation and activates hepatic stellate cells, promoting collagen deposition and ultimately leading to the development and progression of portal fibrosis (13). This continuum from functional impairment to structural damage has been observed in CF-related animal models, such as *CFTR*-knockout rabbits, whose characteristic hepatobiliary lesions closely mirror the pathological changes seen in human CFLD.

It is important to acknowledge a limitation in the molecular diagnosis of this case. The whole-exome sequencing technology employed in this study has limited sensitivity for detecting large deletions, duplications or other structural variations in the *CFTR* gene. Performing a multiplex ligation-dependent probe amplification assay constitutes an important future follow-up step to conclusively exclude the presence of such undetected variants on the second allele and to provide the most comprehensive genotyping. Nonetheless, based on the current findings, c.3139G > T (p.Gly1047Cys) is a definitive pathogenic nonsense mutation and c.1409T > A (p.Val470Glu) is a recognised conditionally pathogenic missense variant. Their combination in trans is considered sufficient to reduce *CFTR* function below the pathogenic threshold. By integrating this with the patient's classic multi-system clinical presentation (pancreatitis, liver disease, respiratory imaging changes), the available evidence strongly supports the diagnosis of atypical CF.

This case highlights the need to consider *CFTR* disorders in children with unexplained abdominal pain and hepatopancreatic lesions. For diagnosis, sweat chloride testing remains first-line where available. In cases with atypical presentation or inconclusive sweat tests, *CFTR* genetic sequencing is invaluable, providing a molecular diagnosis, informing prognosis and genetic counseling, and guiding targeted therapies (6, 19, 22–26).

## Data Availability

The original contributions presented in the study are included in the article/Supplementary Material, further inquiries can be directed to the corresponding author.

## Supplementary material

Supplementary Figure S1 Sanger sequencing of CFTR gene mutation in the family of the patient. A: Sanger sequencing map of the paternal CFTR gene (chr7:117250723 locus). The sequencing peak diagram shows a heterozygous mutation of G > T at the target site. B: Sanger sequencing map of the maternal CFTR gene (chr7:117199534 locus). The peak diagram reveals a heterozygous mutation of T > A at the target site. C: Sanger sequencing map of the patient’s CFTR gene (chr7:117250723 locus). The peak diagram shows overlapping G and T peaks at the target site. D: Sanger sequencing map of the patient’s CFTR gene (chr7:117199534 locus). The peak diagram displays overlapping T and A peaks at the target site.

Supplementary Figure S2 The test results of amylase, lipase, pancreatic amylase and urine amylase for the patient during hospitalization.
