# Supplementary figures and images for: Case Report: Cystic fibrosis transmembrane conductance regulator gene heterozygous variation presenting with abdominal pain and hepatopancreatic lesions in a child
Source: Front Pediatr. 2026 Feb 5;13:1707993. doi: 10.3389/fped.2025.1707993 (PMC12916658; doi:10.3389/fped.2025.1707993)

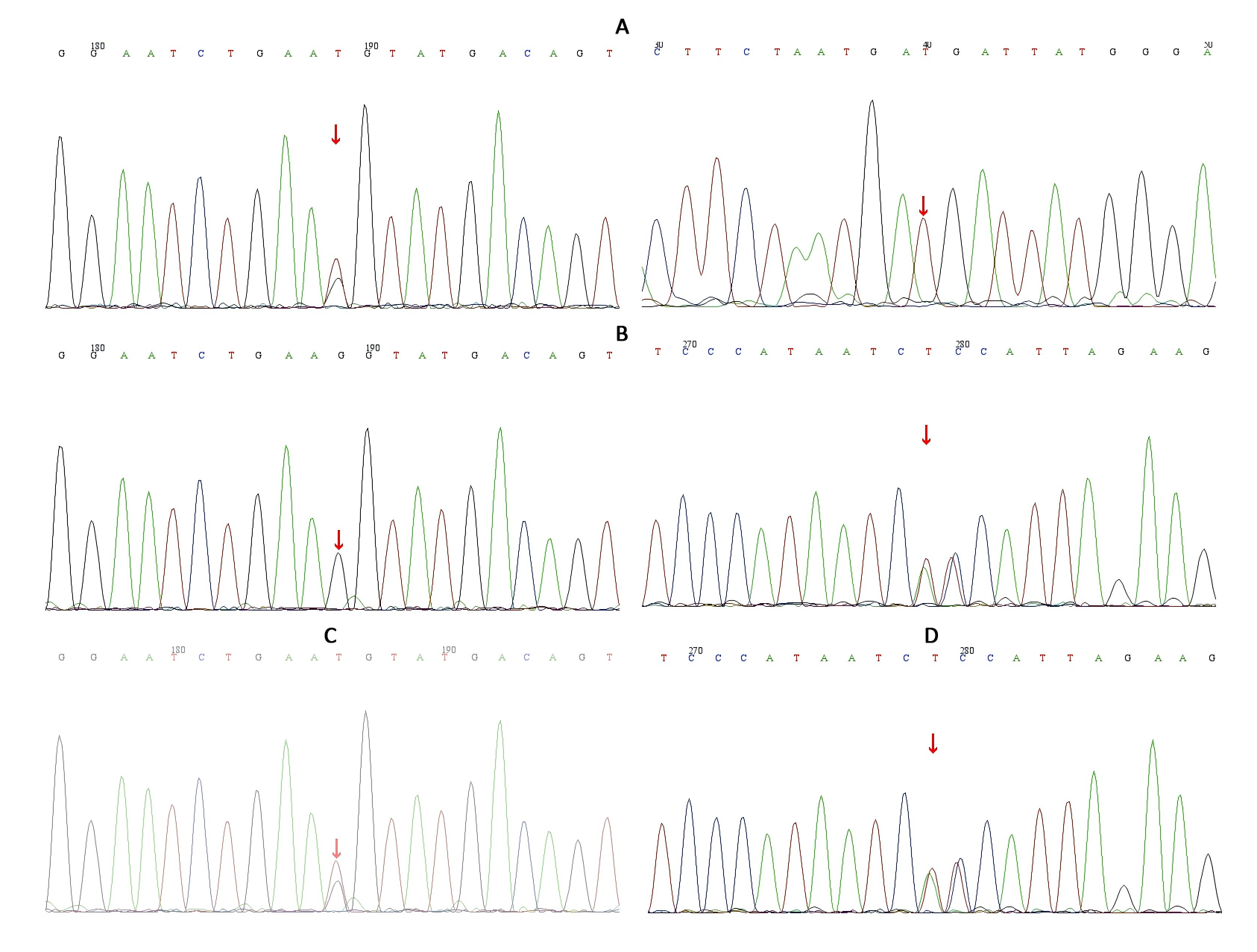

Supplement: Supplementary Figure S1 — Sanger sequencing of CFTR gene mutation in the family of the patient. A: Sanger sequencing map of the paternal CFTR gene (chr7:117250723 locus). The sequencing peak diagram shows a heterozygous mutation of G > T at the target site. B: Sanger sequencing map of the maternal CFTR gene (chr7:117199534 locus). The peak diagram reveals a heterozygous mutation of T > A at the target site. C: Sanger sequencing map of the patient’s CFTR gene (chr7:117250723 locus). The peak diagram shows overlapping G and T peaks at the target site. D: Sanger sequencing map of the patient’s CFTR gene (chr7:117199534 locus). The peak diagram displays overlapping T and A peaks at the target site. [file Image1.tif]

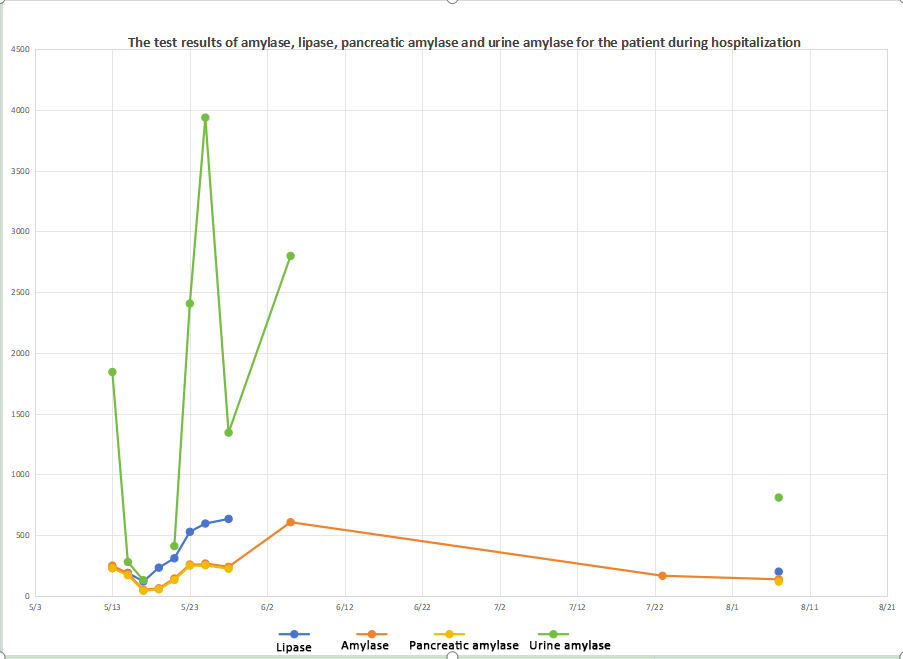

Supplement: Supplementary Figure S2 — The test results of amylase, lipase, pancreatic amylase and urine amylase for the patient during hospitalization. [file Image2.tif]
